# Prognostic Value of A/T/N Biomarkers: Comparing Plasma and Imaging Modalities in Alzheimer’s Disease

**DOI:** 10.1002/alz70861_108113

**Published:** 2025-12-23

**Authors:** Sohyun YIM, Yuna Gu, Jihwan Yun, Sang Won Seo

**Affiliations:** ^1^ Samsung Medical Center, Gangnam‐gu, Seoul Korea, Republic of (South); ^2^ Samsung Medical Center, Sungkyunkwan University School of Medicine, Gangnam‐gu, Seoul Korea, Republic of (South); ^3^ Kyung Hee University Medical Center, Seoul Korea, Republic of (South); ^4^ SAIHST, Sungkyunkwan University, Seoul Korea, Republic of (South); ^5^ Sungkyunkwan University, Suwon Korea, Republic of (South); ^6^ Samsung Medical Center, Gangnam‐gu, Seoul Korea, Republic of (South)

## Abstract

**Background:**

Alzheimer’s disease is characterized by three key pathological features: amyloid‐β plaques (A), tau tangles (T), and neurodegeneration (N), collectively defined as the ATN framework. Compared to imaging biomarkers, the prognostic value of plasma biomarkers remains underexplored, particularly in predicting cognitive decline. This study aims to compare the utility of plasma and imaging biomarkers from the ATN framework in predicting cognitive decline and to evaluate the impact of combining biomarkers across modalities.

**Method:**

We analyzed a cohort of 1614 individuals with varying cognitive statuses, assessing plasma biomarkers (Aβ ratio, *p* ‐tau181, *p* ‐tau231, *p* ‐tau217, NfL) and a sub‐cohort of 130 participants with imaging biomarkers (Aβ PET, tau PET, structural MRI) for their ability to predict cognitive decline. Regression models were used to identify the most predictive markers within each modality and to evaluate the effect of combining ATN biomarkers on prognostic performance.

**Result:**

Tau markers demonstrated the strongest predictive value across both plasma and imaging modalities, with *p* ‐tau217_MSD_ outperforming other plasma biomarkers (adjusted R2= 0.159; *p* <.001) and the neo‐temporal ROI showing the highest predictive power among imaging biomarkers (adjusted R2= 0.354; *p* <.001). Plasma‐based models benefitted from the integration of amyloid and neurodegeneration markers with tau, improving adjusted R² values (adjusted R2= 0.159; *p* <.001, R2= 0.175; *p* <.001, respectively). In contrast, adding additional neurodegeneration markers to combinations of these biomarkers include AT in imaging‐based model decreased the adjusted R² (adjusted R2= 0.361; *p* <.001, adjusted R2= 0.357; *p* <.001, respectively), suggesting that combinations of amyloid and tau PET captures the most relevant prognostic information .

**Conclusion:**

Imaging biomarkers, particularly tau PET, show superior prognostic accuracy compared to plasma biomarkers, whereas plasma biomarkers offer unique advantages in combination models through neurodegeneration markers. These findings underscore the complementary roles of plasma and imaging biomarkers and emphasize the need for tailored strategies for prognostic modeling in Alzheimer’s disease.

